# Intra- and Interobserver Reliability of Bone Volume Estimation Using OsiriX Software in Patients with Cleft Lip and Palate Using Cone Beam Computed Tomography

**DOI:** 10.3390/dj9020014

**Published:** 2021-01-22

**Authors:** Anuraj Singh Kochhar, Maninder Singh Sidhu, Mona Prabhakar, Ritasha Bhasin, Gulsheen Kaur Kochhar, Himanshu Dadlani, Gianrico Spagnuolo, Viral Vijay Mehta

**Affiliations:** 1 Former Orthodontist, Department of Dentistry, Max Hospital, Gurgaon 122001, India; anuraj_kochhar@yahoo.co.in; 2 Department of Orthodontics, Faculty of Dental Sciences, SGT University, Gurugram 122006, India; deanresearch@sgtuniversity.org (M.S.S.); mona.prabhakar@sgtuniversity.org (M.P.); 3 Faculty of Dentistry, University of Toronto, Toronto, ON M5G 1X3, Canada; ritasha.bhasin@mail.utoronto.ca (R.B.); viral484@yahoo.com (V.V.M.); 4 Department of Pediatric & Preventive Dentistry, National Dental College & Hospital, Dera Bassi 140507, India; gulsheenuppal@gmail.com; 5 Department of Periodontology, Kalka Dental College & Hospital, Meerut 210507, India; himdent@hotmail.com; 6 Department of Neurosciences, Reproductive and Odontostomatological Sciences, University of Naples “Federico II”, 80131 Naples, Italy

**Keywords:** cleft lip and palate, unilateral cleft, CBCT, Osirix, bone volume calculation, SABG, alveolar bone grafting

## Abstract

The objective of the current study was to evaluate intra- and interobserver bone volume measurements and effect of orientation on the reliability of bone volume evaluation in as-acquired vis-à-vis oriented cone beam computed tomography scans using Osirix software and possible correlation between gender, age, and bone volume required. For this, 31 cone beam computed tomography (CBCT) scans of 14 girls and 17 boys (aged 9–13) with unilateral cleft lip and/or palate who met the inclusion criteria were analyzed. Efficacy and reliability of third party software Osirix for bone volume calculation was assessed using as acquired and oriented volumes by three specialists (a radiologist, an orthodontist, and an oral maxillofacial surgeon). The dataset and readings were anonymized to prevent any bias. Two-way mixed model analysis on as-acquired and oriented observations exhibited intra-class coefficient (ICC) values ≥ 0.90. Wilcoxon signed rank test (*p* = 0.10) and Kruskal–Wallis ANOVA (*p* = 0.46) indicated that although a clinical difference in volume assessment was seen between as-acquired and oriented observations (inter-observer and intra-observer), it was statistically insignificant. Spearman’s bi-variate correlation analysis revealed a significant relation between the type (side) of cleft and bone volume required to fill the defect (*p* < 0.05). Although there was clinical difference in bone volume measurement by the three observers, it was insignificant statistically. Clefts on the left side in the patients had significantly more bone required than the right side, whereas age and gender had no relation with bone needed to fill the defect. OsiriX software provided good reliability in measurements of bone volume.

## 1. Introduction

Secondary alveolar bone grafting (SABG), the technique deployed to augment bone, is an essential treatment method in the management of bony cleft [1,2]. The gold standard for alveolar bone defect repair is autologous bone graft, of which the most widely adopted procedure is Iliac crest bone graft harvesting [3]. The objectives of SABG are maxillary segments’ stabilization, restoration of dental arch continuity, provision of bony support for adjacent teeth, closure of oronasal fistula, improvement in support for alar base, and facilitation of subsequent orthodontic treatment [4,5]. SABG is quintessentially performed at the end of the mixed dentition, prior to permanent canine eruption to provide cromulent periodontal support, albeit any negative repercussions on maxillary growth [6]. Personalized preoperative planning plays a key role in appropriate SABG and includes cognizance of the size as well as the shape of the bone defect in order to aid in an unambiguous evaluation of grafting material required and subsequently a more predictable modus operandi and denouement. Moreover, the preoperative awareness of the bone graft needed may also lead to diminished overall cost; reduced hospital stays; and, more importantly, decreased morbidity [7].

A prerequisite for SABG procedure is a precise assessment of the alveolar defect volume. It helps in procedure preparation, for example, donor site selection and treatment outcome evaluation [8,9,10]. Formerly, the only methods available for alveolar cleft evaluation were conventional two-dimensional (2D) radiography; linear measurements; and subjective evaluations of panoramic, occlusal, and periapical radiographs [11,12].

Nevertheless, two-dimensional radiography has its limitations, such as lack of volumetric information; ambiguous assessment of anatomical structures, owing to enlargement, distortion, or overlap; and deficiencies in landmark discernment, thereby adversely influencing treatment planning and outcomes. Another method used to evaluate ABG outcomes is conventional computed tomography (CT), due to its ability to provide reliable and unerring representations of the anatomical structures and pathological processes [13,14]; however, it is linked with high-dose ionizing radiation exposure, particularly for patients at the developmental age [2,8].

Hence, cone beam computed tomography (CBCT), a three-dimensional method, has been accepted to evaluate the alveolar bone defect. Recently, grafting volume calculation, utilizing CBCT, was found to be reliable [13,15,16]. Surgeons and practitioners can, through volumetric analysis, better comprehend the dental and bony condition in the vicinity of the cleft, assess the amount of bone required for grafting, and inspect the location and quantities of bony bridges formed after the procedure [16]. Studies have endeavored to lend insight into the effect of head orientation on the accuracy of linear measurements but not on the anatomic landmark positions in three dimensions with a change in orientation [17,18,19].

Although a deluge of data is available on the accuracy of landmark plotting and its influence on the orientation of the volume-rendered images, direct evaluation of precision of volume evaluation in patients requiring secondary alveolar bone graft has not been looked into [20]. In the light of such data with uncertain standpoints on the effect of orientation on volume evaluation, we conducted the present study in order to evaluate with the primary objective of intra- and interobserver reliability of bone volume measurements and effect of orientation in as-acquired vis-à-vis oriented cone beam computed tomography scans using Osirix and the secondary objective to find correlation between gender, age, and bone volume.

## 2. Materials and Methods

### 2.1. Subjects

The power analysis and sample size estimation at 80% power, 0.5 alpha level, and large effect size (0.8) revealed that a minimum of 21 patients were required. Therefore, retrospective evaluation of records of 73 North Indian children with non-syndromic unilateral cleft lip and alveolus with or without cleft palate who underwent CBCT scanning 4 weeks prior to SABG surgery were enrolled in the present study, excluding patients with syndromes/mental retardation or with inadequate CBCT image data.

After careful evaluation, 31 CBCT scans of children (Table 1) fulfilling the inclusion criteria were selected for the study. Following the acquisition of CBCT scans using an i-CAT next-generation machine (Imaging Sciences International, Hatfield, Pa (field of view: 17 × 22 cm)), we saved the data in DICOM (version 1.7) format with an isometric voxel size of 0.25, and at window width/level of 3500/1000 HU, the images were reoriented utilizing InVivoDental 5.0 (Anatomage, anatomy imaging software San Jose, CA, USA). CBCT scans were obtained while the subject was sitting upright and in a natural head position.

### 2.2. Measurements and Data Acquisition

**Figure 1:**
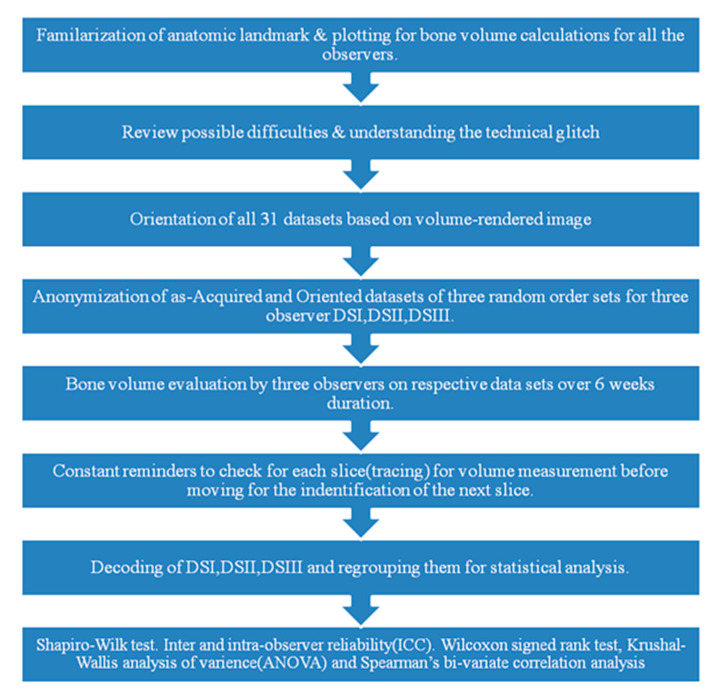
Flowchart representing the methodology.

Three experienced specialists one orthodontist (O1), one radiologist (O2), and one oral maxillofacial surgeon (O3) were solicited to calculate the volume of the cleft region using the Osirix Dicom Viewer (Pixmeo Inc., Genève, Switzerland) (Alonso et al., 2010; Rosset et al., 2004) [1,21]. We used the landmarks given by Linderup et al., which were for outlining the buccal and palatal margins of the alveolar bone defect for buccal/palatal side. For mesial/distal, bone and the alveolar bone defect was defined by the mesial and the distal margins, and the superior/inferior landmarks were from the CEJ of neighboring tooth to the extent of bony deformity. However, in the present study, freehand marking on the axial slices was performed to estimate the bone volume by the inherent feature of the software [20]. The margins of the defect were determined along the buccal/lingual, mesial/distal, and superior/inferior directions, followed by the determination of threshold values for the bone and alveolar bone defect. All 3 observers were offered the same training prior to computing the bone volume, which was an inbuilt characteristic of the software. Furthermore, orientations of the CBCT images were performed by a separate coordinator (C1). Randomization of the data for blinding was performed by another coordinator (C2), who generated 3 random datasets of CBCT, referred to as DSI, DSII, and DSIII for calculating the volume of the cleft region by the 3 observers O1, O2, and O3, respectively (Figure 1).

**Figure 2:**
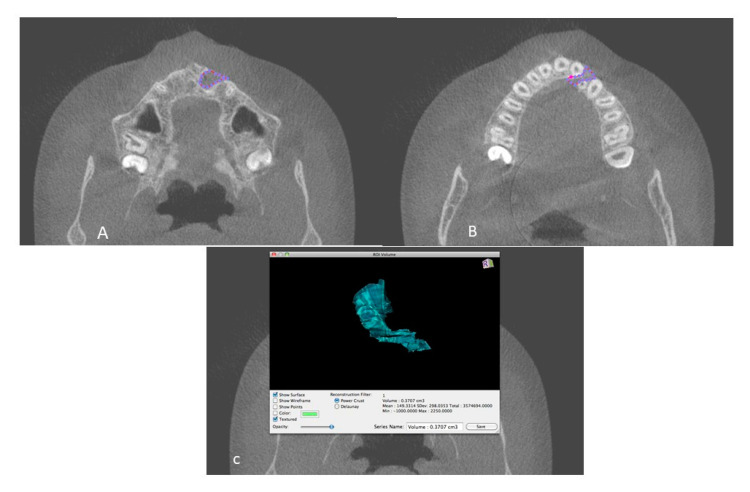
(**A**,**B**) Defect demarcation on the axial slice using drawing tool of OsirixDicom Viewer (Pixmeo Inc.); (**C**) Volume of the defect calculated by Osirix software.

The 3 specialists (O1, O2, O3) who participated in the study were familiarized with each landmark’s boundaries and definitions for tracing the cleft region of interest, and a mutual consensus was achieved. Three anonymized CBCT images were traced. Software fallacies or training was clarified by an expert on the software. Furthermore, the definitions of landmarks for bone volume measurements were refined with the agreement of all experts and any obscurity in landmark localization was resolved via a mutual discussion. (Figure 2).

For the purpose of orientation, the volumes were reoriented by coordinator C1 (who was not involved in the experiment). Volume rotated mediolaterally until the transporionic line of the data became horizontal. Volume rotated until the midsagittal plane of the data oriented vertically, and in sagittal view, the Frankfort plane of the data was oriented horizontally [9]. After orientation, new volumes were acquired and saved. A total dataset of 62 CBCT volumes (31 as-acquired and 31 oriented) were thus created.

### 2.3. Blinding

The datasets created were further anonymized by the coordinator C1 for blinding. For prevention of bias, datasets (as-acquired and oriented) were kept in 1 location and renamed with numbers from 1 to 62 in the order decided by coordinator C2 who had not performed the orientation of the CBCT images. Three random sets (DSI, DSII, DSIII) of data were generated in the same manner for each of the 3 observers (O1, O2, O3). Hence, the observers were neither aware of the orientation of the CBCT datasets nor of the order of the CBCT volumes. Three observers (O1, O2, O3) independently calculated individual volumes of 31 patients 3 times each, over the course of 6 weeks. After bone volume evaluation, the randomized samples were decoded and regrouped into as-acquired and oriented datasets for analysis.

### 2.4. Statistical Analysis

Statistical analysis for the present study was performed using Statistical Package of Social Sciences (SPSS) version 20.0. Normality of data was assessed using the Shapiro–Wilk test. Intra-class coefficient (ICC) was reviewed to assess inter and intra-rater reliability. Due to the non-normal distribution of data, we performed the Wilcoxon signed rank test in order to evaluate differences between as acquired and orientated values amongst the different observer groups. The Kruskal–Wallis analysis of variance (ANOVA) was applied to as-acquired and oriented observations in order to check for differences between the findings of three observers. Spearman’s bi-variate correlation analysis was performed to ascertain the influence of age, sex, and type(side) of cleft of the study subjects on as-acquired and reoriented observations. Level of statistical significance was established at *p* < 0.05.

### 2.5. Ethical Considerations

Following the approval of the study from the institutional ethical committee of the institution, vide approval number SGTDC/PPL/Com./E.C./14Aug2010, we conducted the current study at the Department Orthodontics and Dentofacial Orthopedics, Faculty of Dentistry, SGT University, India, from March 2011 to May 2013. Assurance regarding the confidentiality was given to the patients after the research objectives were explained to the patients who volunteered for the study.

## 3. Results

A total of 31 subjects were enrolled for the present study, of which 14 (45.2%) were girls and the remaining 17 (54.8%) were boys. All the patients had a unilateral type(side) of cleft, with about 58% exhibiting cleft on the right side and the remaining 42% on the left side. Age range of study subjects was 9 to 13 years, with the mean observed at 11 ± 0.98 years (Table 2).

**Figure 3:**
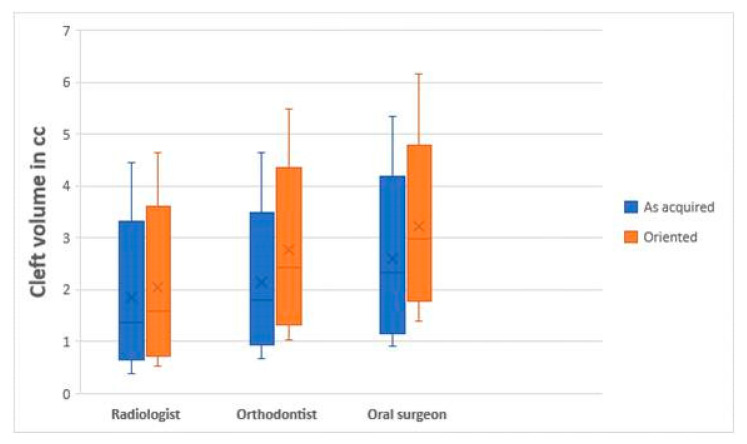
Box and whisker plot exhibiting observations of various specialists.

Two-way mixed model analysis on as acquired and oriented observations exhibited ICC values ≥0.90 within observers(intra) and ≥0.80 amongst the observers(inter). As-acquired and oriented data sets exhibited non-Gaussian distribution as per the Shapiro–Wilk test, which is also depicted in the box and whisker plot (Figure 3).

**Figure 4:**
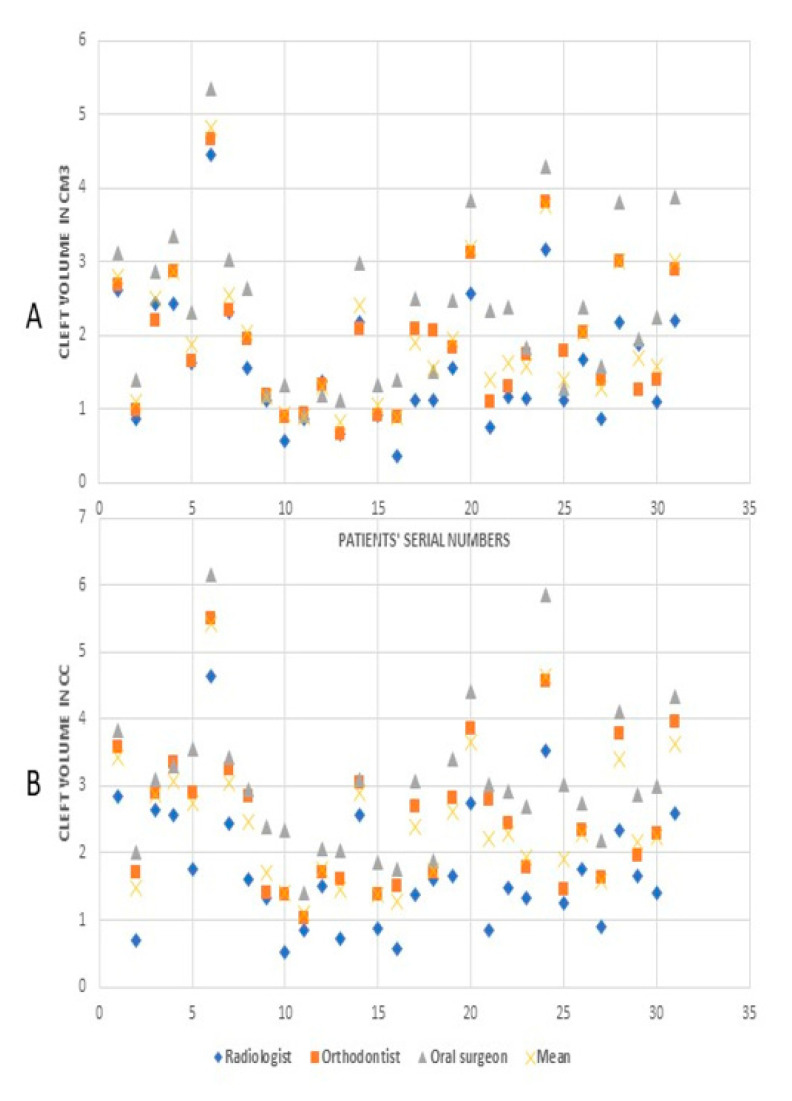
Cleft volumes by different observers: (**A**) values from as-acquired volume, (**B**) values from oriented volumes.

The group-scatter graphs represent bone volumes for every study subject as assessed by the O1, O2 and O3. The as-acquired and oriented observations by three different observers are showcased in different group-scatter graphs (Figure 4). A collective mean for every study subject generated on the basis of observed values by the three observers is also highlighted in the graphs.

The Wilcoxon signed rank test indicated that the clinical difference in volume assessment between as-acquired and oriented observations amongst the three observer groups was not statistically significant (*p* = 0.10). Moreover, the appraised volume differences between as-acquired and oriented observations across observers were not found to be statistically significant as per the Kruskal–Wallis ANOVA (*p* = 0.46). Spearman’s correlation analysis revealed a significant influence of the side of cleft on as-acquired and oriented observations across observers (*p* < 0.05) (Table 2). This was comprehended by the observation that the volume of bone for the unilateral cleft on the left side was consistently higher as compared to the unilateral cleft on the right side (Table 3).

## 4. Discussion

Alveolar cleft is a frequently encountered hereditary condition. An integrated comprehension of the morphology and volume of the bone defect is essential for meticulous secondary alveolar bone grafting [2,13] The most frequently used grafting procedure, utilizing the iliac crest, has the benefit of harvestation of substantial amounts of bone graft. However, while extensive harvest should be avoided, inadequate grafting may lead to failure. Hence, a customized approach is imperative [3].

There is no consensus in data regarding the importance of stage of root development on graft success, yet some authors suggest that the ideal timing for SABG is when the unerupted canine is close to the cleft border and the root is half to two-thirds developed. Moreover, in a study by Oberoi et al., there was no significant difference observed in SABG prognosis between canine root development [8,20,22]. On the contrary, according to Vandersluis et al., better results and lesser unfavorable effects were observed in pre-canine eruption SABG, in contrast with post-canine eruption SABG, thus justifying the age group for the sample as 9–13 years for the current study [23].

Despite the critical role played by the amount of bone required for reconstruction of the bone defect, rather than using objective criteria, surgeons mostly determine the amount of graft on the basis of their experience, potentially resulting in either superfluous or scarce graft harvest. Heiser et al. (2004) were the first to attempt measurement of palatal volume, and this was done indirectly by weight correlation [24]. According to Quereshy et al., objective criteria to estimate the amount of graft were landmarks and linear calculations, corresponding to the cleft width, height, and facial-palatal length. However, this technique had the drawback of an upward bias or overestimation [2,25]. Nevertheless, evaluation of the cleft side can now be performed by software analysis of a 3D image to permit pre- and post-assessment of the alveolar defect, thereby aiding in scrupulous treatment planning and outcome evaluation [8]. Therefore, in the present study, we used a third-party software, OsiriX, to calculate bone volume and evaluate its reliability for the same in UCLP patients by three experienced observers [21].

The OsiriX software has various advantages, such as customizability, owing to its flexible user interface. Furthermore, it can be customized for various clinical applications or specialties, albeit the requirement of auxiliary programs or software. Alonso et al. evaluated the objective parameters (such as bone volume, height, labiolingual anatomy, and bone morphology) through CT and the use of OsiriXDicom Viewer (Pixmeo Inc.) and arrived at the conclusion that superlative precision and enhanced image quality were observed [1]. Comparable results were seen in the present study, and non-significant differences were obtained when using the OsiriX software, ensuring relative ease in understanding, verifying reliability, and showing good reproducibility of the bone volume calculations by the three different observers (O1, O2, and O3).

Comparative studies are arduous to conduct, in the absence of explicit definitions. Moreover, reproducibility is essential to determine validity; therefore, unambiguous criterions are essential for utilizing any technique of three-dimensional alveolar bone defect assessment [20]. Many methods have been published in the literature for bone volume evaluation, such as water displacement technique [2,15], free hand tracing [1], subtraction method [13], 3D printing [10,26], and computer engineering [27]. In the present study, we used the landmarks given by Linderup et al., which were modified by free hand marking of the relevant slices and estimating the bone volume. Since three different authors were trained about the methods and similar results were obtained both in as-received and oriented volumes, our study showed good understandability, precision, and validation of the reproducibility of the technique.

No generically accepted standard protocols are followed for utilizing head-positioning devices such as head straps, chin, and upper lip rests [19]. During 2D and 3D analyses with natural head position, there are chances of head movement owing to the long scanning time [28]. Although there are a multitude of studies evaluating the landmark errors in plotting in as-acquired and oriented images, there is a paucity of studies calculating the volume of using the same methodology. Hence, in the current study, reliability of as-acquired and oriented images was assessed. Weber et al. [28] observed statistically insignificant differences in points marked in either of the three planes of space, whereas mean angular deviation in reference planes was significant with maximum reproducibility in coronal view, followed by axial and minimum in sagittal view. Similar results were obtained by Hassan et al. [18]. These findings were in accordance with the present study. However, significant variation was observed by Cevidans et al., concluding that head orientation may not only affect reliability but relative location of anatomy [17].

Regarding the evaluation of precise measurement of the volume of the cleft, Sezgin et al. commented that in their study, the 0.2 mm slice-thickness group had the highest asymptotic significance value (*p* = 0.6) [29]. Although slice thickness up to 1 mm can be selected for volume computations on CBCT images, the most accurate values are discerned when utilizing slices with minimal thickness. Another study by Molen et al. suggested that more pertinent for such studies would be using small voxel sizes, also reducing the effect of partial volume averaging [30]. In the current study, 0.25 mm slice thickness was used, which is the minimum possible thickness required for the software to join and make a volume its inherent feature with least standard deviation. This was similar to Kasaven et al. [26], who used 0.2 mm voxel size. Previous studies by Oberoi et al. [22] used axial slices of 0.4 mm while Feichtinger et al. [31,32] utilized 1.5 mm thickness. On the other hand, Honma et al. [33] and Alonso et al. [1] made use of larger sizes, with these being 2 mm and 1 mm slice thickness, respectively [14].

In the present study, the evaluation of the unilateral cleft volume of age 9–13 years old revealed as-acquired mean volume by a radiologist was 1.6138 + 0.88 cm^3^, orthodontist was 1.9089 + 0.93 cm^3^, and oral and maxillofacial surgeon was 2.3831 + 1.08 cm^3^. Although a considerable clinical variation in their readings was shown, there was no statistical difference observed as compared to post-orientation mean volumes of 1.7619 + 0.94 cm^3^, 2.55 + 1.06 cm^3^, and 3.05 + 1.08 cm^3^, respectively. Linderup et al. suggested CBCT to be appropriate for reporting preliminary normative volumetric data due to the highly reproducible evaluation of volume [20]. Weber et al., in their systemic view, said that bone volume calculation generally falls by 4.11% (downward bias) when I-cat machines are used for evaluation, making the utilization of supplementary bone essential [14]. Oberoi et al. [22] in their study had a mean bone volume of 0.61 cm^3^ preoperatively, whereas it was 1.40 + 0.37 mL in the study by Chen et al. [13] and 1.1 + 0.3 cm^3^ by Honma et al. [33]. According to Chou et al., mean volume of the sample in unilateral alveolar cleft defect reconstructions was 1.0 mL, while in bilateral reconstructions it was 2.0 mL [10,34]. This was further backed by various studies [15,16,22,35]. In the study by Barbosa et al. [34], the mean defect volume was 0.86 cm^3^ (range 0.34 to 1.97 cm^3^). Numerous studies found the preoperative defect size to range from 1.3 to 2.1 cm^3^, which was in accordance with the cleft volumes we calculated [36,37,38].

As per recent systematic reviews, no technique is considered a gold standard for evaluation of alveolar cleft defects. Furthermore, there is a lack of unanimity considering various 3D imaging-based modalities for the same [20,33]. Good reliability of volume calculation was obtained in the present study in intra-observer and inter-observer measurements, which was in accordance with Linder up et al. and Barbosa et al., who concluded similar findings [20,34]. In our study, although the area of expertise of all the three observers was different, the findings suggest that OsiriX software is easy to grasp and can provide repeatable results. Although the marking of volumes was clinically varied, the variation was not potent enough to show any significant readings. In a study by Oberoi et al. [22], there was consensus between the two raters (inter-observer) and within a single rater (intra-observer), with Pearson correlation coefficient of above 0.9 for both, which was in conjugation to our findings. The intra-observer agreement value according to the ICC was 0.97 (with a 95% CI ranging from 0.85 to 0.99), demonstrating excellent agreement in relation to the ICC interpretation [34].

Our study demonstrated that, as compared to the right-side cleft, the left-side unilateral cleft required more volume of bone, which was statistically significant. In our comparison, age and gender did not have any significant influence on the bone volume. These findings were contrary to the findings of Chen et al., who concluded that alveolar defect volume was significantly affected by gender and cleft type (*p* < 0.01) [13]. This is in agreement with previous studies, suggesting that maxillary development is usually attained by 11 years in the transverse as well as sagittal planes. Furthermore, these studies found significant differences in alveolar cleft defect volume between males and females. This phenomenon can be explained by many cephalometric analyses, which show that females have smaller mid-facial lengths compared with males [39,40]. Although awareness that these factors can influence alveolar cleft defect volume could be of some assistance to surgeons, it is not possible to estimate the amount of bone needed for alveolar bone grafting in terms of these factors alone.

### Limitations

In the current study, bilateral alveolar cleft patients were not included. Furthermore, due to the lack of standard benchmarks for acquiring and reconstructing images, systematic protocols for analysis of alveolar bone defects utilizing CBCT are inadequate. Moreover, consensus has not been observed with anatomical boundary selection by various authors [7,22,41]. Hence, methodological comparison of such studies is challenging, and custom boundaries were considered for this study, which is not ideal. Moreover, the volumes estimated could not be validated after the surgical treatment.

## 5. Conclusions

- Although there was clinical difference in bone volume measurement by the three observers, it was insignificant statistically.
- Clefts on the left side in the patients had significantly more bone required than the right side, whereas age and gender had no relation with bone needed to fill the defect.
- OsiriX software provided good reliability in measurements of bone volume, proving to be a promising tool for valuable clinical information according to treatment protocol.

## Figures and Tables

**Table 1:** Demographic data and cleft distribution.

Variable(s)		*n*	%
Age	9	6	19.4
	10	11	35.4
	11	7	22.6
	≥12	7	22.6
Gender	Girls	14	45.2
	Boys	17	54.8
Type (side)of cleft	Unilateral left	13	41.9
	Unilateral right	18	58.1

**Table 2:** Spearman’s correlation analyses of socio-demographic patterns and as-acquired and oriented observations.

	As-Acquired	Oriented	Gender	Age	Type (Side) of Cleft
As-acquired	-	0.97 *	−0.20	0.18	0.20 *
Oriented		-	−0.16	0.23	0.17 *
Gender			-	−0.88	0.01
Age				-	−0.14
Type of cleft					-

* Significant finding.

**Table 3:** Mean bone volume for unilateral cleft on left and right side.

**Unilateral Cleft on Left Side (*n* = 13)**								
	**As-Acquired**				**Oriented**			
	**Radiologist**	**Orthodontist**	**Oral Maxillofacial Surgeon**	**Collective Mean**	**Radiologist**	**Orthodontist**	**Oral Maxillofacial Surgeon**	**Collective Mean**
Mean	1.93	2.18	2.66	2.26	2.12	2.79	3.29	2.73
Median	1.87	2.07	2.46	1.95	1.66	2.81	3.10	2.62
SD	1.09	1.15	1.31	1.16	1.11	1.31	1.44	1.27
**Unilateral Cleft on Right Side (*n* =18)**								
	**As-Acquired**				**Oriented**			
	**Radiologist**	**Orthodontist**	**Oral Maxillofacial Surgeon**	**Collective Mean**	**Radiologist**	**Orthodontist**	**Oral Maxillofacial Surgeon**	**Collective Mean**
Mean	1.37	1.70	2.18	1.75	1.49	2.37	2.88	2.25
Median	1.14	1.70	2.27	1.58	1.39	2.31	2.96	2.22
SD	0.62	0.69	0.86	0.69	0.71	0.84	0.73	0.72

## Data Availability

The data presented in this study are available on request from the corresponding author. The data are not publicly available due to institutional/ethical restrictions.
